# Metastases to the Thyroid Presenting as a Metabolically Inactive Incidental Thyroid Nodule with Stable Size in 15 Months

**DOI:** 10.1155/2014/643986

**Published:** 2014-04-10

**Authors:** Subhashini Yaturu, Raina A. Patel

**Affiliations:** ^1^Stratton VAMC, Albany, NY 12208, USA; ^2^Albany Medical College, Albany, NY 12208, USA

## Abstract

Though the thyroid gland has a rich vascular supply, incidence of metastatic disease from distant organs is rare. Here we present an unusual case of metastases to the thyroid with several interesting features. A 63-year-old male with history of adenocarcinoma of the right lobe lung (5 years prior to presentation), treated with surgery and chemotherapy, followed by new adenosquamous lung cancer in the left lobe of the lung (one year prior to presentation), treated surgically followed by adjuvant chemotherapy, was referred to Endocrinology section for evaluation of an incidental thyroid nodule on CT chest. Ultrasound (US) of the thyroid revealed a complex, predominantly hypoechoic lesion measuring 1.8 cm within the lower pole of the right thyroid lobe and a subcentimeter lesion in the left lobe of the thyroid. Review of prior CT chests showed that the lesion in the right lobe was stable for 15 months, with no evidence of a hypermetabolic lesion on PET scan. The subcentimeter lesion was not seen on prior CT scans. US guided fine needle aspiration (FNA) and pathology of the lobectomy of the thyroid confirmed adenosquamous carcinoma. Interesting features in this case are that the thyroid metastases occurred without any evidence of synchronous lesions elsewhere, the size was stable over 15 months, and the lesions were metabolically inactive.

## 1. Background

Clinically relevant metastatic neoplasms to the thyroid gland are rare. The incidence of metastasis to the thyroid gland in autopsy series varies from 1.25% to 24% in patients with widespread malignant neoplasms. Secondary thyroid tumors may be accompanied by synchronous metastatic lesions to other organs, for example, to the lungs. In a 43-case clinical review, the location of the primary tumor in secondary tumors to thyroid is reported to be as follows: kidney as the most common primary tumor site (33%), followed by lung (16%), breast (16%), esophagus (9%), and uterus (7%) [1]. A recent literature review reports that the most common non-thyroidal malignancies that metastasize to the thyroid include in the order of frequency are renal cell carcinoma (48%), colorectal cancer (10.4%), lung cancer (8.3%), breast cancer (7.8%), and sarcoma (4.0%) [2]. Among 619 patients operated for thyroid malignancies, Ménégaux and associates reported that metastases to thyroid from non thyroidal origin was as high as 2.2% [3]. A recent case report includes pancreatic cancer metastasizing to thyroid [4]. Here we present an interesting case with secondary tumor to thyroid without synchronous lesions elsewhere and with stable size for 15 months.

## 2. Clinical Case

In August 2012, a 63-year-old Caucasian male was referred to the Endocrinology Section for evaluation of a thyroid nodule. In October 2007 he had T2N0M1 (AJCC 6th edition) non-small-cell lung cancer. He had resection of the upper lobe of right lung, and histology was consistent with adenocarcinoma. In addition, he was treated with 4 cycles of carboplatin/gemcitabine, which was said to be the standard at that time (could not receive cisplatin/navelbine because of diabetic neuropathy). He was followed with CT scans of the chest. In July 2010, he had recurrence of the mass in the right upper lobe, in the area of the scar, and had a wedge resection which again showed adenocarcinoma. Since it was rT1a, he did not receive adjuvant chemotherapy at that time.

In August 2011, he was found to have another lesion in the left upper lobe of the lung and underwent left VATS upper lobectomy with mediastinal lymph node dissection and intercostal nerve block and pleural biopsy. Surgical pathology found adenosquamous carcinoma which was considered a new primary pT1a N1 cM0 (AJCC 7th edition). He again received 4 cycles of adjuvant chemotherapy with carboplatin/gemcitabine with neupogen support.

**Figure 1:**
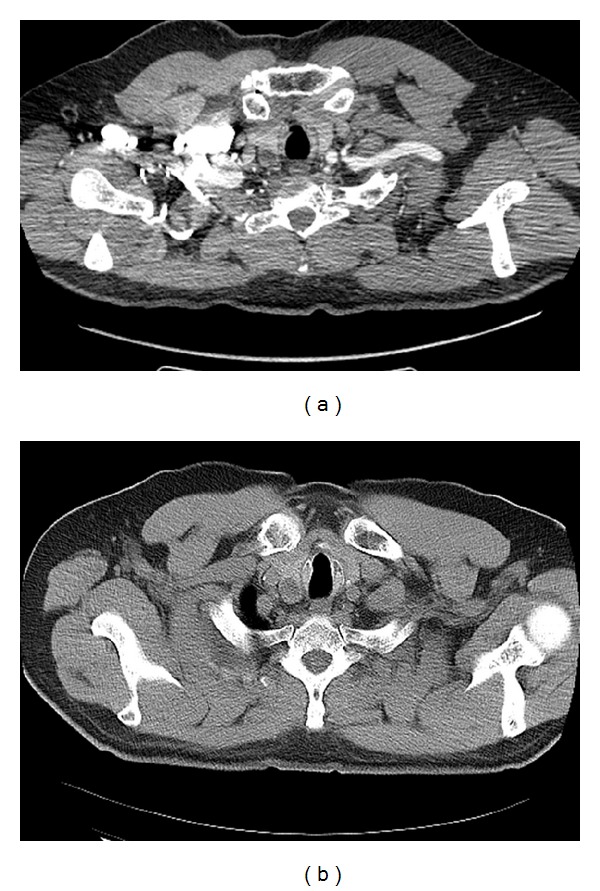
CT chest: thyroid nodule in the neck: (a) on 26 March, 2011; (b) on 24 July, 2012.

**Figure 2:**
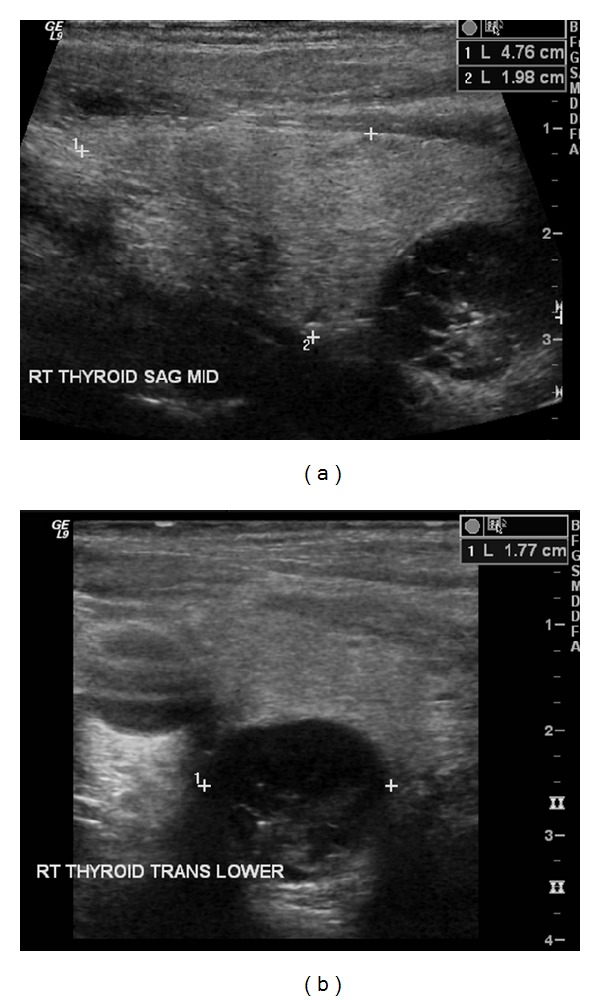
US thyroid: 1.8 cm hypoechoic lesion. (a) Sagital view and (b) transverse view of right lobe.

Follow-up CT scans of the chest revealed the 1 cm thyroid nodule as shown in Figure 1, but a PET CT done in August 2011 showed no uptake in the thyroid. Ultrasound of the thyroid done on 3 August, 2012, showed a 1.8 cm hypoechoic lesion in the right lobe of the thyroid. US guided FNA of the thyroid nodule on 15 October, 2012, by the endocrinologist showed scant atypical cells, suspicious for malignancy, as shown in Figure 2.

**Figure 3:**
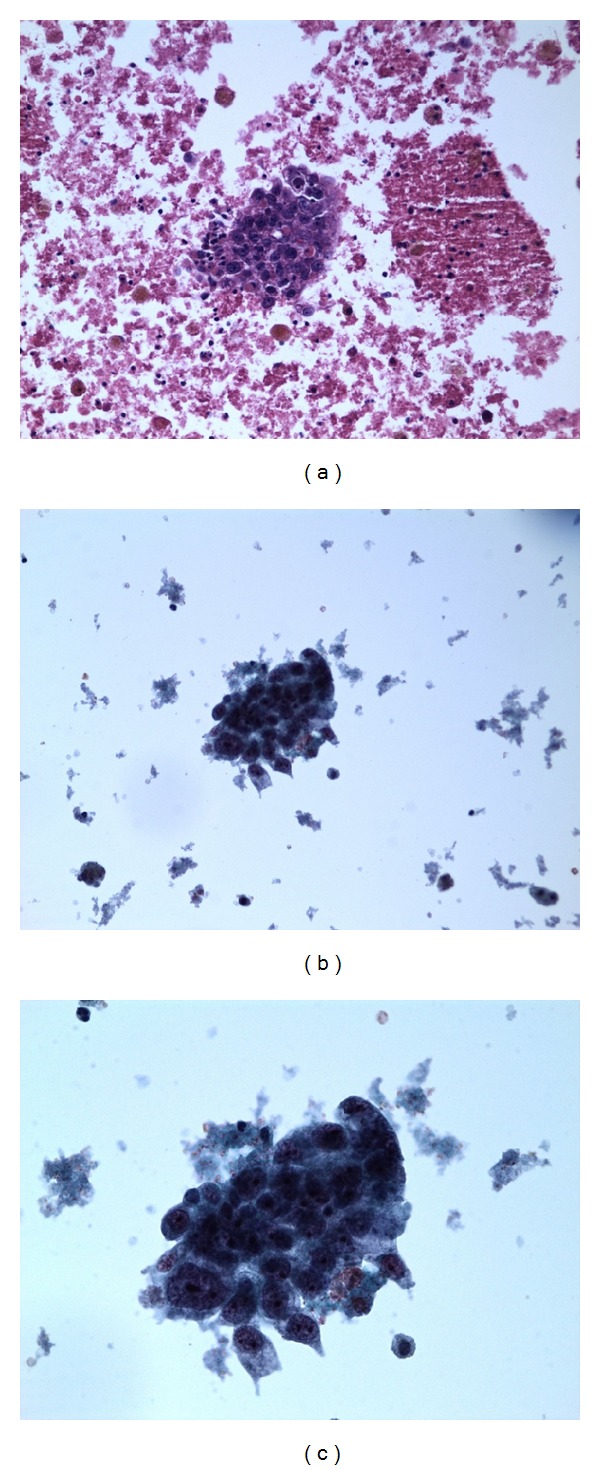
FNA thyroid nodule of right lobe of thyroid. (a) Cell block prep. 20x. (b) Liquid based prep. 20x. (c) Liquid based prep. 40x.

**Figure 4:**
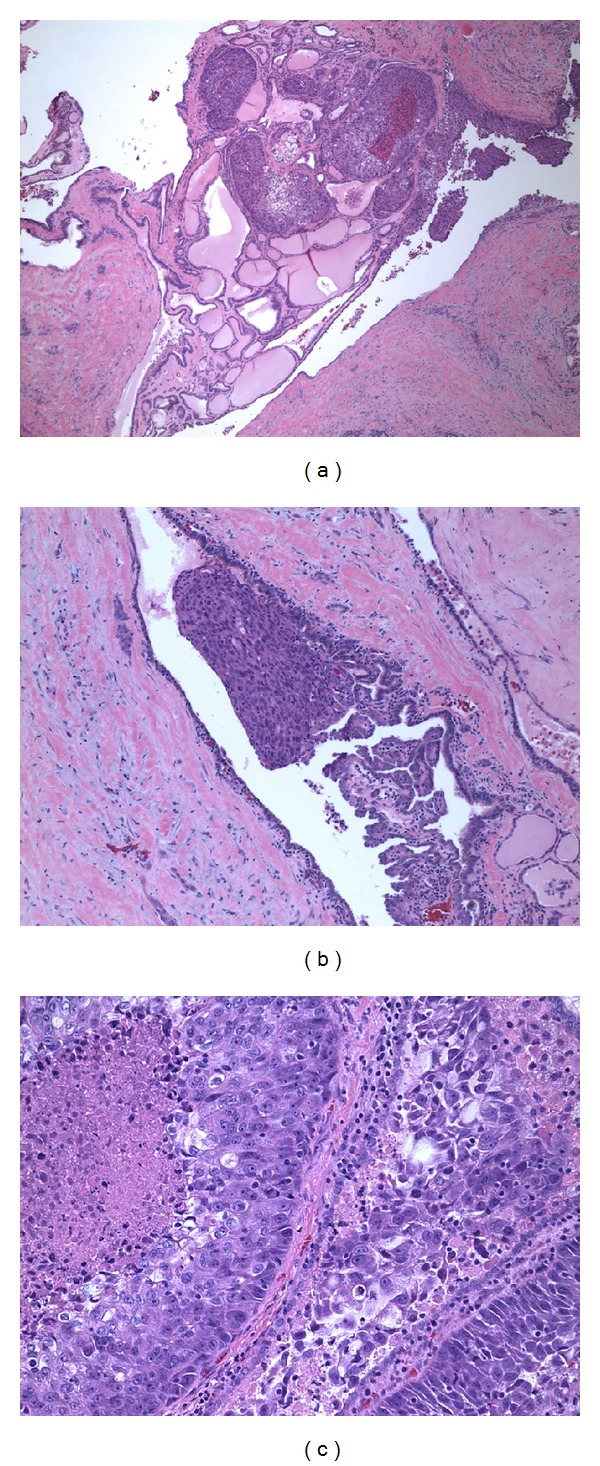
Adenosquamous carcinoma involving adenomatoid nodule. (a) 4x. (b) 10x. (c) 20x.

On 28 November, 2012, the patient underwent right-sided thyroid lobectomy, though total thyroidectomy was suggested. Surgical pathology confirmed adenosquamous cell metastasis from his lung cancer (diagnosed in 2011) (Figure 4). Margins were negative. The slides were reviewed in JPC and confirmed. A restaging PET scan on 14 December, 2012, showed uptake related to surgical changes with no evidence of disease. Pathology results are shown in Figures 3 and 4 below. The left lobe was resected later, as the nodule in the left lobe was increasing in size and pathology confirmed benign nature of the lesion.

## 3. Imaging Studies

*(1) CT Scans of Chest.* CT scans of chest related to thyroid nodule showed the following: on 24 July, 2012 (Figure 1(b)): 1.3 × 1.5 cm. hypodense nodule within the lower pole of right lobe thyroid; on 12 April, 2012 (Figure 1(a)): a 1.6 cm hypodense nodule within the lower pole of right lobe thyroid (previously 1.2 cm); on 26 September, 2011: a 1.2 cm nodule within the lower pole of right lobe thyroid, stable compared to prior study; on 20 March, 2011: a one cm hypodensity within the lower pole of right lobe thyroid.

*(2) PET CT Chest.* On 28 June, 2011: the neck demonstrates normal uptake in the cervical lymph nodes. The thyroid is enlarged and heterogeneous in density but negative on PET.

*(3) Ultrasound Thyroid.* On 3 August, 2012: the right lobe of the thyroid measured approximately 4.8 × 2.0 × 2.2 cm. A complex, predominantly hypoechoic lesion measuring 1.8 cm was seen within the lower pole of the right thyroid lobe (Figure 2). Lack of halo and lack of intranodular calcification were noted with no significant intranodular vascularity. The left lobe of the thyroid measured approximately 4.5 × 2.0 × 1.3 cm. A complex, predominantly hypoechoic lesion was seen within the mid-pole of the left thyroid lobe measuring up <1 cm.

## 4. Discussion

There are several interesting features in this case. First and foremost is that there was no significant interval increase in size of the thyroid nodule on CT scans of chest in 15 months. There are no reported cases of similar nature. Since the lesion was present at the time of the second malignancy (it is seen in the March 2011 CT, Figure 1(a)), the chemotherapy for the adenosquamous carcinoma may have decreased the rapidity of growth of the metastatic lesion. The second interesting feature is that there is no uptake on PET with F-18 deoxyglucose (FDG) in the thyroid nodule, indicating that the metastatic lesion was metabolically inactive. PET with F-18 deoxyglucose (FDG) is a useful technique for the characterization of pulmonary nodules to distinguish between benign and malignant lesions and is considered the most accurate imaging modality for the assessment of nodal and distant metastases from lung cancer [5]. PET/CT was reported to have sensitivity, specificity, positive and negative predictive values, and accuracy for the detection of malignant extra pulmonary lesions 92, 98, 89, 98, and 97%, respectively [6]. On a practical level, a PET-negative study may allow conservative approach and avoidance of unnecessary invasive procedures as negative predictive value is considered as 100% specific [7]. False negative results are rare and said to occur in small (<10 mm) nodules due to partial volume effect or the effect of respiratory blurring, or in some subtypes of lung malignancy with a low intrinsic FDG avidity, such as adenocarcinoma in situ. It is considered that pulmonary lesions with visually absent uptake indicate that the probability of malignancies is very low [7–9]. In general, integrated FDG PET/CT is considered significantly better than stand-alone CT for lung cancer staging [10, 11]. In a recent review of F-18-FDG-PET/CT thyroid incidentaloma is considered as a relevant clinical finding; diffuse uptakes and most focal uptakes are said to be commonly caused by benign diseases, whereas about one third of focal uptakes are malignant; the most frequent malignant histological type responsible for F-18-FDG-PET/CT thyroid incidentaloma is said to be papillary thyroid carcinoma [12]. The other interesting feature in this report is that there are no synchronous metastases anywhere else. Thyroid metastases usually occur when there are metastases elsewhere (synchronous), sometimes many years after the diagnosis of the original primary tumor, and show poor prognosis in general [13].

The diagnosis in this case was made on a fine needle aspiration of the thyroid nodule. In a retrospective study of fine needle aspirations and metastases, Kim and associates reported that FNA biopsy confirmed metastatic disease in 19 patients; thyroid metastases were found during the initial workup of thyroid nodules in eight patients, and in 14 patients the interval from diagnosis of primary tumor to the detection of thyroid metastasis varied from 8 months to 15 years, with a median of 54 months [13]. Certain sonographic features can help in distinguishing between benign and malignant nodules. Benign features are being well defined, cystic, and thinly septated, having mostly peripheral vascularity and containing colloid or dense or dysmorphic calcifications. Malignant features include being ill defined, irregular, necrotic, and locally invasive and having significant intranodular vascularity. Moreover, certain sonographic features can be used to stratify risk for malignancy. The most accurate features significantly associated with malignancy were reported to be posterior acoustic shadowing (87%), many diffuse calcifications (82%), rim calcifications (81%), and taller than wide shape (79%). The subjective level of suspicion was said to have correlated well with the presence of malignancy (76%) [14]. The distinction between primary and secondary malignant thyroid tumors by clinical examination and imaging can be challenging as there are no specific features except for the history of malignancy.

## 5. Conclusion

Though the thyroid gland can be an uncommon site of metastases, one should consider and rule out secondary tumor especially in a patient with a prior history of malignancy, even when the nodule was metabolically inactive by PET scan or showed insignificant interval change.
